# Supplementary material for: Induced Human Regulatory T Cells Express the Glucagon-like Peptide-1 Receptor
Source: Cells. 2022 Aug 19;11(16):2587. doi: 10.3390/cells11162587 (PMC9406769; doi:10.3390/cells11162587)
Supplement: Supplementary file 1 [file cells-11-02587-s001.zip › cells-1835665-supplementary.pdf]

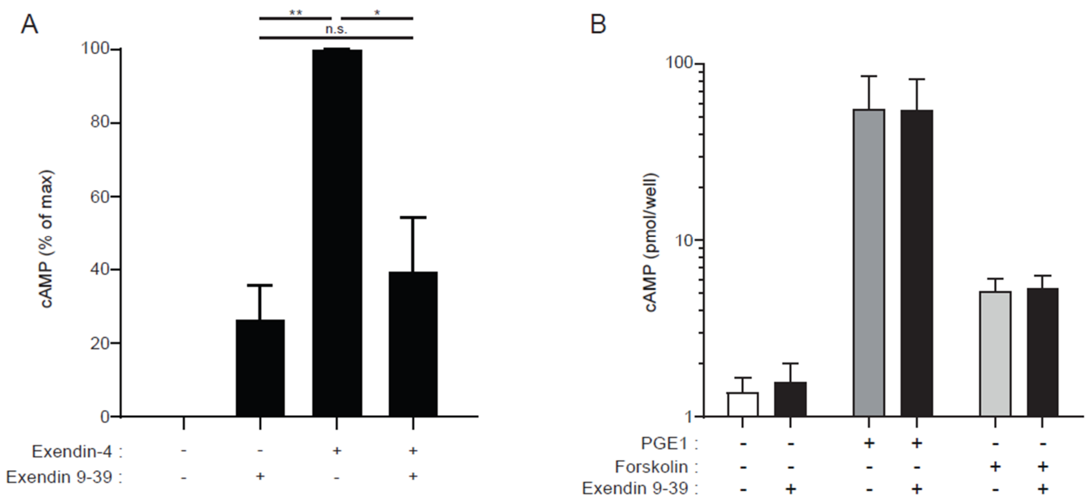

Figure S1. Exendin 9-39 specifically inhibits cAMP accumulation induced by exendin-4. (**A**) Relative cAMP accumulation in CD4+ T cells activated in Th2 medium in the presence of 100 nM 25(OH)D3 untreated or treated with 0.1 µM exendin-4 in the presence or absence of 10 µM of the GLP-1R antagonist exendin 9-39. Data are normalized to values in the absence of exendin-4 and exendin 9-39 (mean + SEM, n = 3, ordinary 1-way ANOVA with post hoc test (Tukey's), *p < 0.05, **p < 0.005). (**B**) cAMP accumulation in CD4+ T cells activated in Th2 medium in the presence of 100 nM 25(O)HD3 and treated with 0.1 µM PGE1 or 1 µM forskolin in the presence or absence of 10 µM exendin 9-39 (mean + SEM, n = 2).

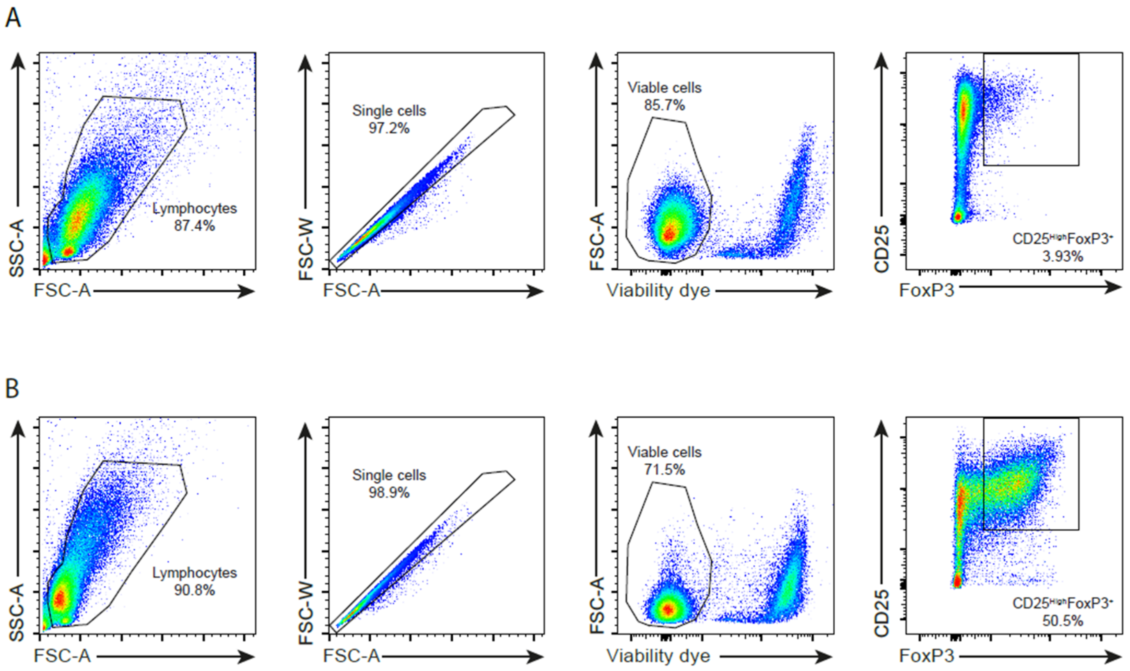

Figure S2. Gating strategy for iTreg cell detection. Gating strategy used to determine the percentage of CD25hiFoxP3+ cells of CD4+ T cells activated for 120 h. Representative examples of cells cultured in the absence of differentiation factors (Th0, **A**) and in Treg cell differentiation medium (**B**) are shown. The percentage of cells within each gate is indicated.

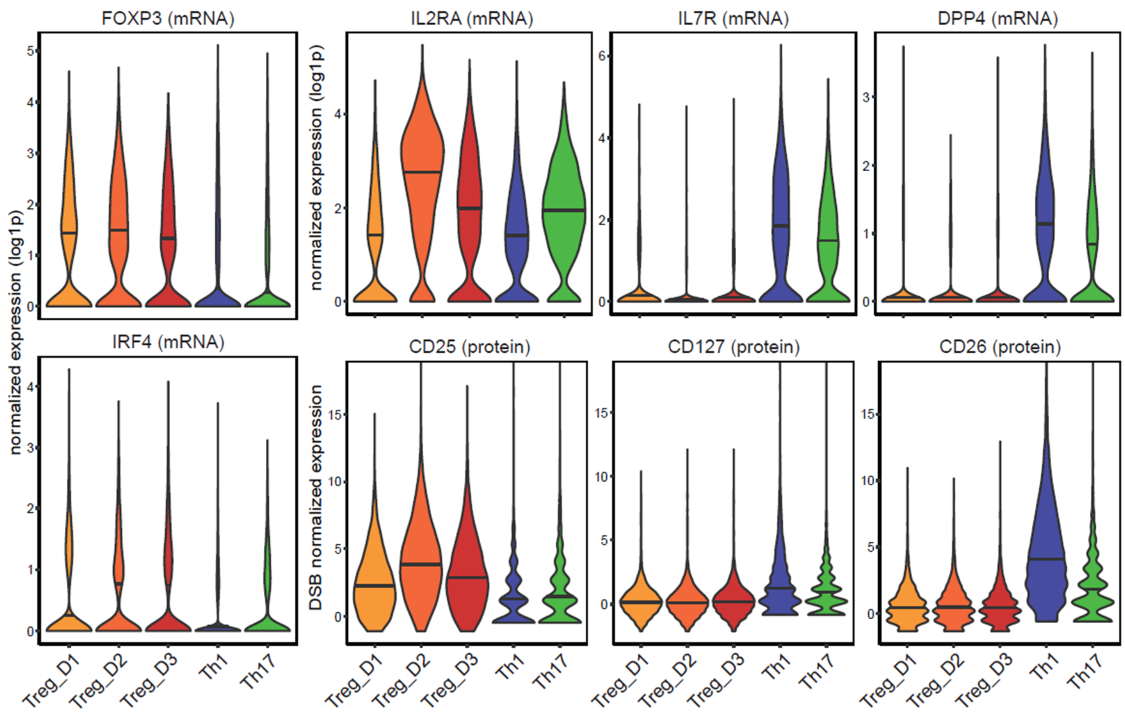

Figure S3. Violin plots of selected markers. Expression of FOXP3, IL2RA, IL7R, DPP4 and IRF4 mRNA as well as CD25, CD127 and CD26 protein of iTreg cells from donor 1-3 and Th1 and Th17 cells as indicated.

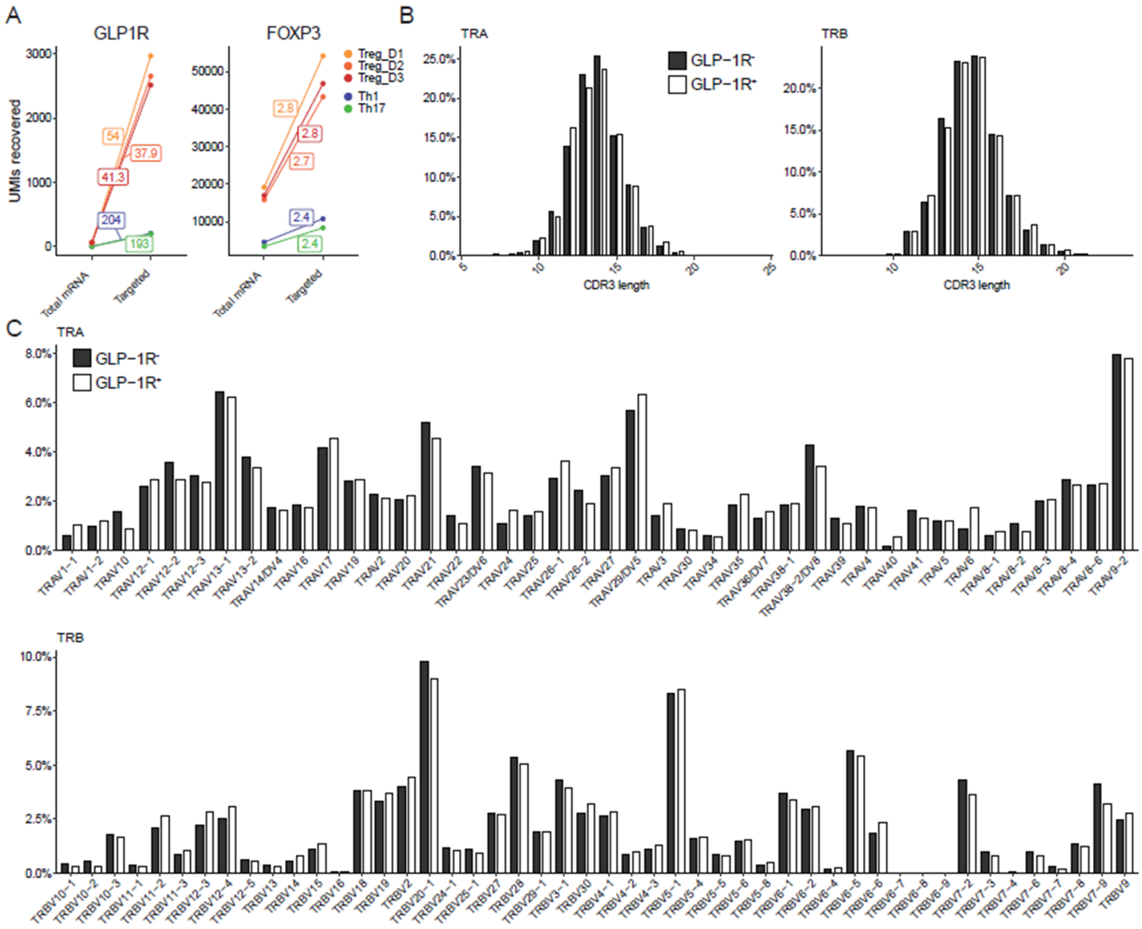

Figure S4. The GLP-1R is not restricted to distinct TCR sequences. (**A**) Comparison of GLP-1R expression using unique molecular identifier (UMI) recovery from whole transcriptome mRNA library (non-enriched) and through nested PCR-based enriched (targeted) libraries. Colors denote donor and numbers show fold-change from non-enriched to enriched detection. (**B**,**C**) Comparison of T cell receptors between GLP-1R- (black) and GLP-1R+ (white) cells showing (**B**) complementarity-determining region (CDR) 3 length of the TRA (left) and TRB (right) rearranged receptor chains as well as (**C**) V-segment usage of the TRA (top) and TRB (bottom) loci.

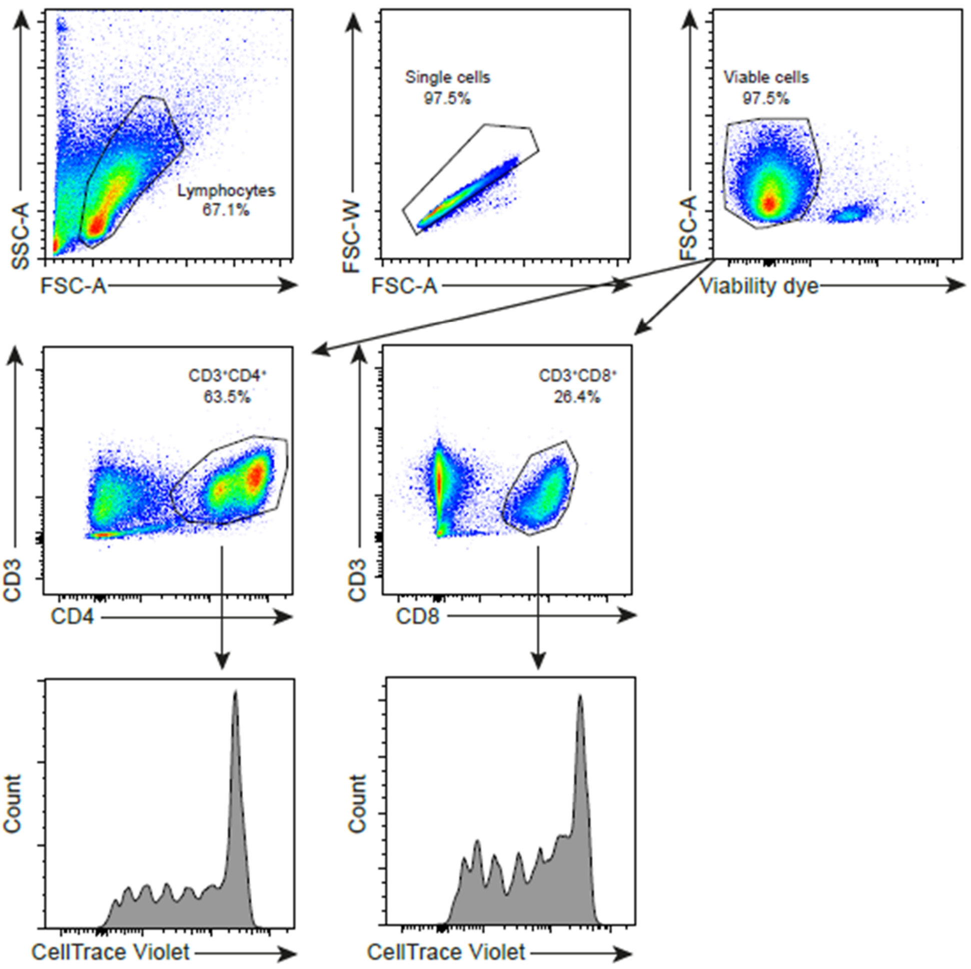

Figure S5. Gating strategy for analyses of viable and proliferating cells. Live lymphocytes were gated based on size, complexity and staining with a viability dye (top panel). CD4+ and CD8+ T cells were identified by double expression of CD3 and CD4 or CD8, respectively (middle panel), and their proliferation index determined from the histograms shown in the lower panel by use of FlowJo's proliferation platform. The percentage of cells within each gate is indicated for one representative donor.

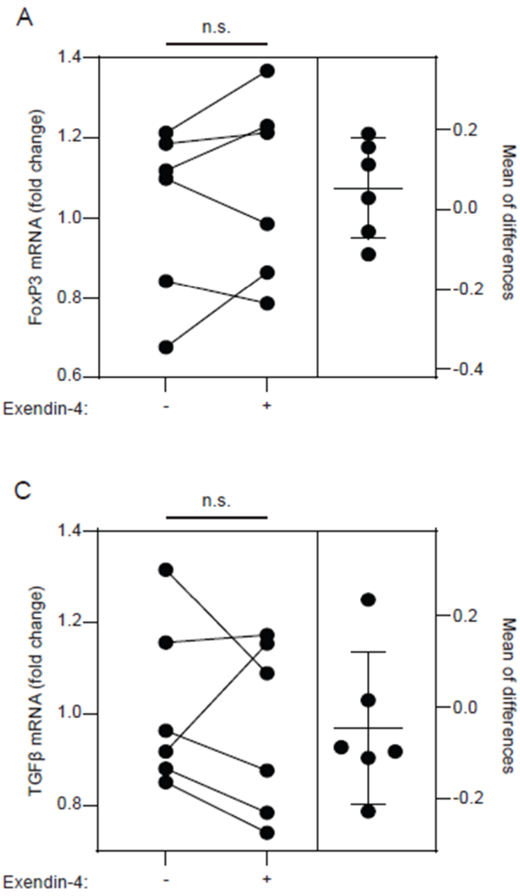

Figure S6. FoxP3, CD25 and TGFβ expression in iTreg cells. FoxP3 (**A**), CD25 (**B**) and TGFβ (**C**) expression in CD4+ T cells activated for 120 h in Treg polarizing medium and subsequently not treated or treated with exendin-4 (10 nM) for 24 h as indicated (estimation plot, n = 6, Student's t-test, (paired, two tailed)).

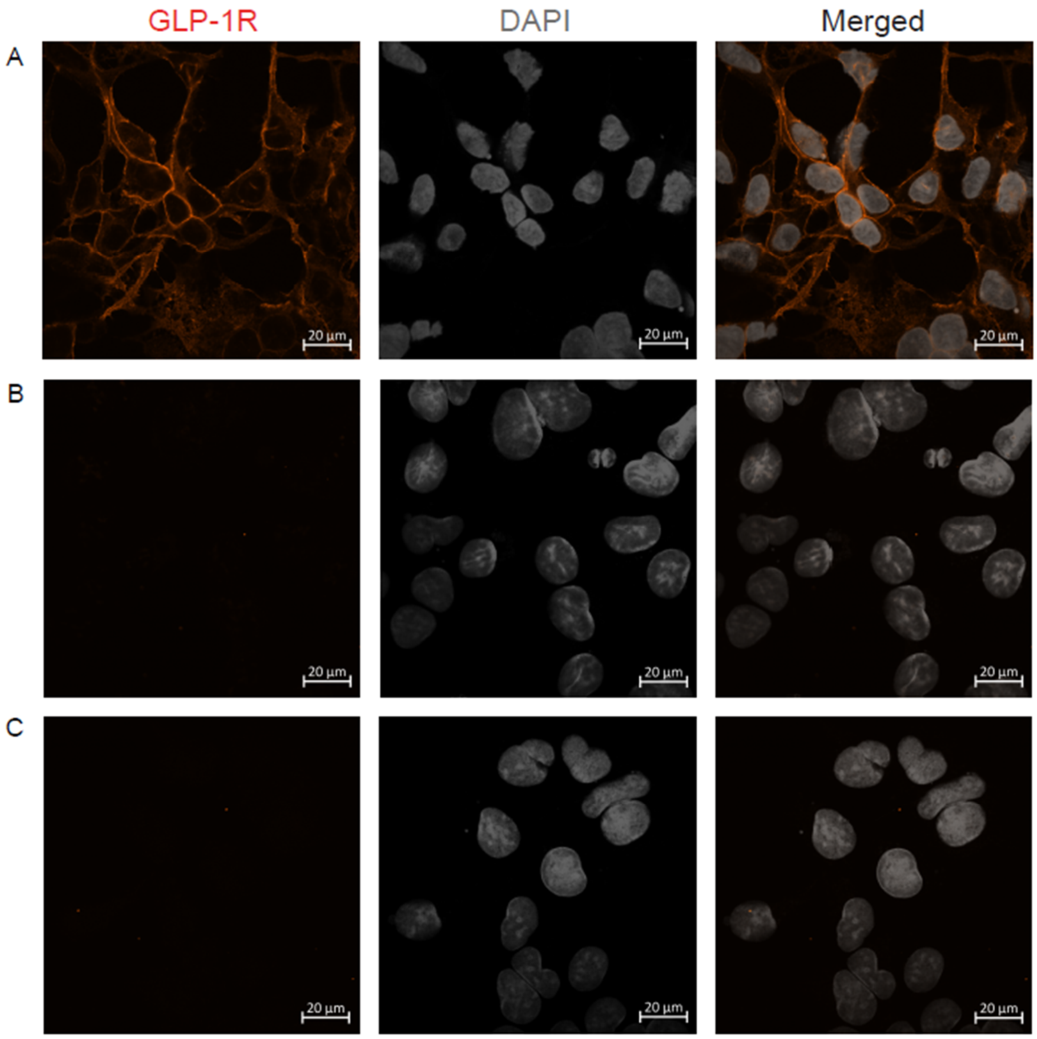

Figure S7. GLP-1R control staining. First column GLP-1R (red), second column DAPI (grey) and third column merged stained fluorescent microscopy images of (**A**) transfected GLP-1R^{+} HEK293 and (**B**) GLP-1R^{-} WT HEK293. (**C**) WT HEK293 only stained with secondary goat anti-rabbit AF555 Ab and DAPI.
